# Outcomes after resection of primary cardiac sarcoma

**DOI:** 10.1016/j.xjon.2021.08.038

**Published:** 2021-09-03

**Authors:** Saad M. Hasan, James Witten, Patrick Collier, Michael Z. Tong, Gosta B. Pettersson, Nicholas G. Smedira, Andrew Toth, Dale Shepard, Eugene H. Blackstone, Eric E. Roselli

**Affiliations:** a Department of Thoracic and Cardiovascular Surgery, Heart, Vascular, and Thoracic Institute, Cleveland Clinic, Cleveland, Ohio; b Cardio-Oncology Center, Heart, Vascular, and Thoracic Institute, Cleveland Clinic, Cleveland, Ohio; c Department of Cardiovascular Medicine, Heart, Vascular, and Thoracic Institute, Cleveland Clinic, Cleveland, Ohio; d Department of Quantitative Health Sciences, Research Institute, Cleveland Clinic, Cleveland, Ohio; e Department of Hematology and Oncology, Oncology Institute, Cleveland Clinic, Cleveland, Ohio

**Keywords:** cardiac sarcoma, malignant cardiac tumor, 3D, 3-dimensional, R0, microscopically negative resection margin

## Abstract

**Objective:**

To evaluate the outcomes of surgical resection of malignant primary cardiovascular tumors.

**Methods:**

From 1983 to 2018, 32 patients underwent surgical resection of malignant primary cardiovascular sarcoma at Cleveland Clinic. Mean age was 48 ± 15 years, and 19 (59%) were women. Outcomes are compared between those with complete resection and those without, and in relation to primary location.

**Results:**

The most common histologic subtypes were angiosarcoma (n = 8 [25%]) and high-grade undifferentiated sarcoma (n = 7 [22%]). Fourteen (44%) involved the left heart, 9 (28%) the right heart, 8 (25%) the pulmonary arteries, and 1 (3%) the aorta. There was clinical evidence of isolated extracardiac metastases in 8 (25%). Six (19%) patients were deemed unresectable at surgery, undergoing biopsy and palliative debulking followed by referral for definitive chemotherapy and/or radiation. The remaining 26 (81%) patients underwent 31 tumor resections with curative intent. Seven (22%) patients had previously undergone a resection or biopsy at another institution. There were 10 second-time resections, 2 third-time resections, 1 fourth-time resection, and no operative mortalities. Median survival was 3 years, with estimated survival at 6 months and 1, 5, and 10 years of 90%, 73%, 31%, and 17%, respectively. Of the 8 (25%) who were considered disease-free following surgery, 4 experienced recurrences during follow-up.

**Conclusions:**

Primary cardiac sarcoma continues to be a challenging disease with poor prognosis. Aggressive resection with curative intent, frequent surveillance for local and distant recurrence, and systemic and local multimodality treatment optimizes outcomes.

**Figure:**
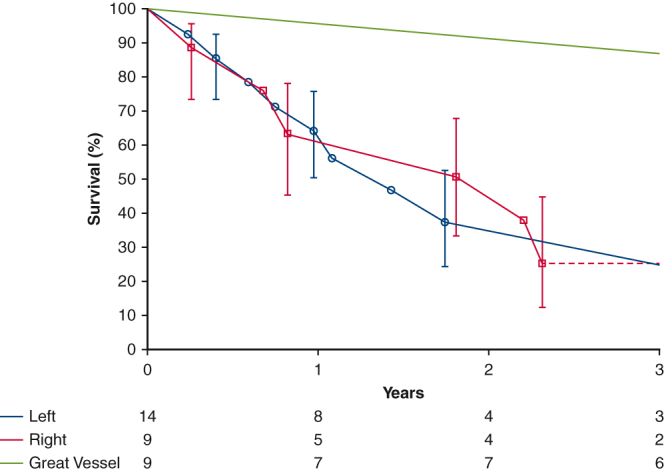
Survival is poor in patients with cardiovascular sarcoma regardless of tumor location.

**Central Message**

Primary cardiac sarcomas carry a poor prognosis. Surgical resection, rigorous postoperative surveillance, and aggressive multimodality treatment of recurrences are keys to prolonging survival.

**Perspective**

Primary cardiac sarcoma is a rare condition afflicting young patients, with median survival of 3 years. Surgical resection remains the mainstay of treatment and modern imaging techniques may reduce the occurrence of incomplete resection. Rigorous surveillance for commonly occurring, local, and distant recurrence is crucial, because reoperations and systemic treatments may prolong survival.

See Commentary on page 391.

Malignant primary cardiovascular tumors are a rare and challenging clinical problem with a dismal prognosis often afflicting young patients during the fourth and fifth decades of life. The prevalence of primary cardiac tumors is 0.02% in autopsy series with 25% being malignant, of which 75% are sarcomas.^1^^,^^2^ Median survival in published series ranges from 9 to 33 months.^3^, ^4^, ^5^, ^6^, ^7^, ^8^, ^9^, ^10^, ^11^, ^12^, ^13^, ^14^, ^15^, ^16^ Most are clinically silent until an advanced stage, and often considered unresectable due to proximity to critical structures. However, improved imaging and surgical techniques have allowed for more complex resections, and when microscopically negative (R0) resection can be achieved, there is a clear survival benefit.^5^, ^6^, ^7^, ^8^, ^9^, ^10^, ^11^ The addition of neoadjuvant therapy may also contribute to better achievement of R0 resection and survival.^11^, ^12^, ^13^, ^14^ We analyzed the outcomes of resection and the influence of multimodality treatment in managing patients with primary malignant cardiac sarcoma at a tertiary center.

## Patients and Methods

### Patients

**Figure 1:**
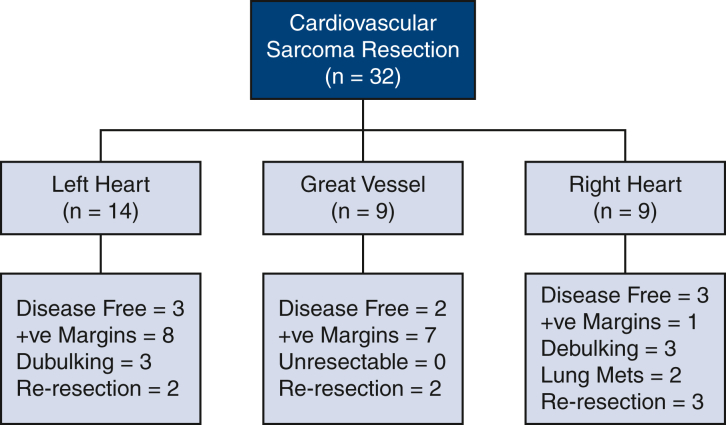
Summary of surgical treatment strategies and outcomes of resection of cardiovascular sarcoma. *+ve*, Positive.

From 1983 to 2018, 32 patients underwent surgical resection of a malignant primary cardiovascular sarcoma at Cleveland Clinic. Patients were excluded if tumor pathology was benign, or if the tumor was a metastasis from a noncardiac source. This study focused on patients undergoing surgery for cardiovascular sarcomas (Figure 1). Mean age of patients with these tumors was 48 ± 15 years, and 59% were women (Table 1). Of the 32 sarcomas treated with surgery, 9 (28%) originated from the right heart, 14 (44%) from the left heart, 8 (25%) from the pulmonary arteries, and 1 (3%) from the aorta (Table 1). The most common histologic subtypes were angiosarcoma (n = 8 [25%]) and high-grade undifferentiated sarcoma (n = 7 [22%]). There was clinical evidence of isolated extracardiac metastases in 8 (25%).

**Table 1:** Sarcoma patient characteristics and outcomes (N = 32)

Variable	n∗	Result
Age	32	48 ± 15
Female	32	19 (59)
Comorbidity		
Previous cardiovascular surgery	31	10 (32)
Congestive heart failure	31	5 (16)
Chronic obstructive pulmonary disorder	31	3 (10)
Previous stroke	31	4 (13)
Previous smoker	30	14 (47)
Location		
Right atrium	32	9 (28)
Right ventricle	32	3 (9)
Superior vena cava	32	4 (13)
Left atrium	32	17 (53)
Left ventricle	32	0
Pulmonary artery	32	8 (25)
Aorta	32	1 (3)
Postoperative outcomes		
Operative mortality	32	0
Stroke	31	1 (3)
Reoperation for bleeding	31	0
Prolonged ventilation (>24 h)	30	5 (17)
Need for dialysis	30	1 (3)
Disease-free (R0 resection + no distant disease)	32	8 (25)

Values are presented as n (%) or mean ± standard deviation.

∗ Patients with data available.

Six (19%) patients were deemed unresectable in the operating room and underwent biopsy with palliative debulking followed by definitive chemotherapy and/or radiation. The remaining 26 (81%) patients underwent 31 tumor resections with curative intent. Seven (22%) patients had previously undergone incomplete resection with biopsy at another institution and underwent reoperation with an attempt at complete resection at our institution. Additionally, there were 10 second-time resections, 2 third-time resections, and 1 fourth-time resection that occurred during follow-up of the initial operations. Other patient details are included in Table 1.

### Clinical Data

Data were retrieved from the prospective Cleveland Clinic Cardiovascular Information Registry, with additional information extracted from medical records. Data included demographic characteristics, medical (cardiac and noncardiac) comorbidities, and echocardiographic variables. The Cleveland Clinic Institutional Review Board approved the use of the data for research, with patient consent waived.

### End Points

The primary clinical end point was all-cause mortality. Secondary end points included tumor recurrence and postoperative complications as defined for the Society of Thoracic Surgeons National Cardiac Database.^17^

### Treatment

#### Surgical technique

Details of the surgical technique varied depending on location of the tumor and extent of resection. The main goal of treatment was complete resection with curative intent for most, but not all patients. Most tumors were approached via median sternotomy using cardiopulmonary bypass and cardiac arrest when the tumor invaded the cardiac chambers and resection of cardiac structures was needed to achieve complete resection. A debulking approach may be relevant in some patients with severe inflow or outflow obstruction on the right side, or with a space occupying lesion causing tamponade, but even in those situations we aim for as complete resection of the tumor as possible. Bovine pericardial patch material was used to reconstruct the atrial free wall in 9 patients, and the ventricular free wall in 3 patients. Valves and great vessels were reconstructed with standard commercially available prosthetics and allografts as necessary, including bioprosthetic mitral valve replacement in 7 patients and tricuspid valve replacement in 1 patient, pulmonary allograft replacement in 5 patients, the use of annuloplasty rings for mitral valve repair in 4 patients and tricuspid valve repair in 2 patients, and proximal aortic reconstruction with a polyethylene terephthalate graft in 1 patient.

#### Medical therapy

Our treatment approach has evolved and improved over time. Patients are currently reviewed by a multidisciplinary team made up of surgeons, cardiologists with an interest in cardio-oncology, medical oncologists with an interest in sarcoma, radiation oncologists, and cardiovascular imaging specialists. Each case is reviewed by the team to discuss potential diagnoses, need for biopsy, indications for pre- and postoperative adjuvant therapy (eg, chemotherapy and radiation) and the indications for surgery. Excellent imaging has allowed for a more complete assessment of critical structures at risk and preparation to deal with those issues by modifying the exposure and understanding the plans for reconstruction at the time of operation.

### Data Analysis

Short- and long-term survival and recurrence were assessed nonparametrically by the Kaplan-Meier method. All analyses were performed using SAS statistical software version 9.4 (SAS Institute Inc, Cary, NC).

### Presentation

Continuous variables are summarized by mean ± standard deviation and categorical variables are summarized by frequencies and percentages.

## Results

There were no operative mortalities. Complications included stroke in 1 (3%), prolonged ventilation in 5 (17%), blood product transfusion in 18 (58%), open chest with delayed closure in 1 (3%), and permanent pacemaker implantation in 1 (3%). Sixteen (50%) patients received adjuvant chemotherapy, consisting of doxorubicin and ifosfamide dual therapy in most. Ten (31%) received adjuvant radiation therapy.

**Figure 2:**
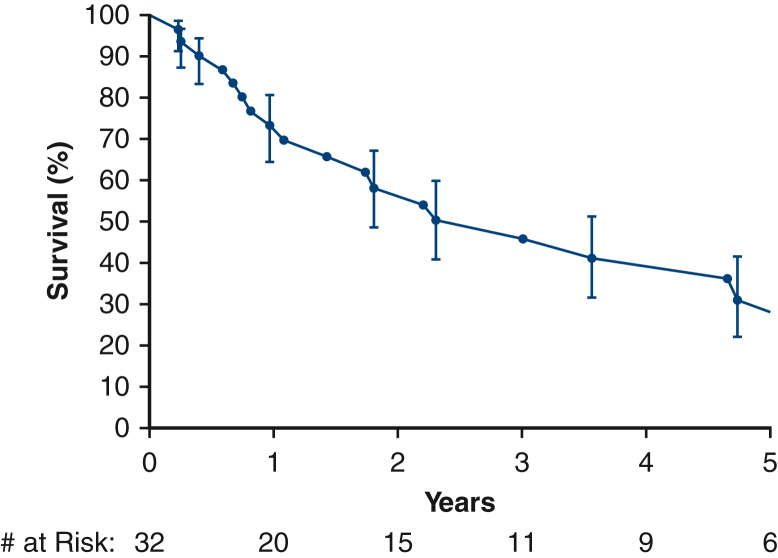
Survival estimated by Kaplan-Meier method after cardiovascular sarcoma resection, with a median survival of 3 years. *Each symbol* represents a death, and *vertical bars* 68% confidence intervals equivalent to ±1 standard error. Number of patients remaining at risk is shown along the horizontal axis.

**Figure 3:**
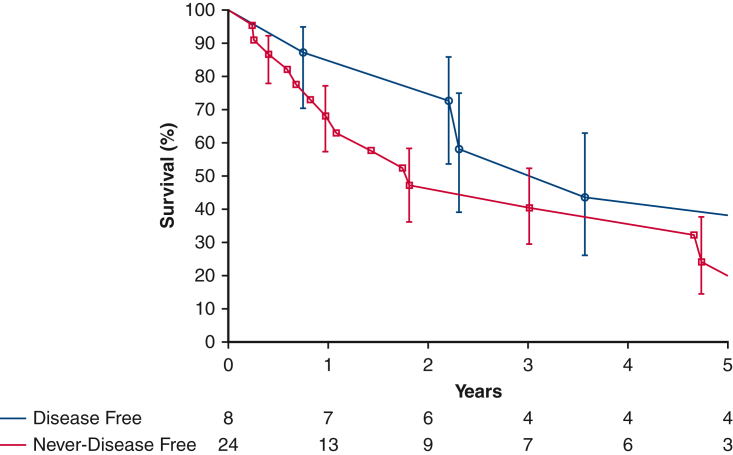
Survival estimated by Kaplan-Meier method in patients who were disease-free immediately following surgical resection of cardiovascular sarcoma versus those who were never disease-free. *Each symbol* represents a death, and *vertical bars* 68% confidence intervals equivalent to ±1 standard error. Number of patients remaining at risk is shown along the horizontal axis.

Median survival time was 3.0 years (95% confidence interval, 1.08-4.7 years), with estimated survival at 6 months and 1, 5, and 10 years of 90%, 73%, 31%, and 17%, respectively (Figure 2). Eight (25%) patients were considered disease-free following surgery, having undergone an R0 resection and having no evidence of distant metastases at the time of surgery. Of these, 4 experienced recurrences during follow-up (Figure 3) at a median time of 3 years.

### Right Heart Sarcoma

Nine patients with right heart sarcoma underwent 12 surgical resections (Figure 1). Operative techniques for the primary surgery included right atrial resection in 1 patient (11%), right ventricular resection in 1 patient (11%), combined right atrial and right ventricular resection in 2 patients (22%), right atrial and superior vena caval and innominate vein resection in 2 patients (22%), and palliative debulking in 3 patients (33%). Three patients (33%) were deemed disease-free postoperatively. Three patients (33%) were considered unresectable intraoperatively, 2 patients were found to have new pulmonary metastases, and 1 patient was debulked and left with gross positive margins.

Adjuvant chemotherapy alone was employed following 4 tumor resections, and combined adjuvant chemoradiotherapy was used in 1 patient. Two local recurrences were treated with definitive chemotherapy.

Of the initially disease-free patients, 1 developed 2 recurrences during follow-up, which were treated with repeat resection and adjuvant chemotherapy the first time, and definitive chemotherapy the second time. The second disease-free patient underwent a redo resection for the first recurrence, chemotherapy for a second recurrence, and a third operation for surgical debulking for tumor progression with outflow obstruction. The third disease-free patient was lost to follow-up. Of the 6 never-disease-free patients, 3 underwent biopsy or debulking due to unresectability. Of the 2 with pulmonary metastases at presentation one survived 3 months, and the other was lost to follow-up overseas. One patient had microscopically positive margins, and was lost to close follow-up after initial adjuvant chemotherapy.

### Left Heart Sarcoma

Fourteen patients had a left heart sarcoma and underwent 16 operations (Figure 1). Seven patients (50%) had previously undergone an attempted resection elsewhere. Surgical techniques for the primary operation included left atrial resection in 4 patients (29%), left atrial resection with mitral valve repair in 3 patients (21%), left atrial resection with mitral valve replacement in 3 patients (29%), and palliative debulking in 3 patients (21%). Three patients (21%) were disease-free postoperatively, 8 patients (57%) had positive margins, and 3 patients (21%) were deemed grossly unresectable.

Of the disease-free patients, 2 did not have close follow-up, and 1 died at 9 months of a gastrointestinal complication. Patients with positive margins underwent multiple treatments for disease progression. For local progression, 1 patient underwent re-resection with adjuvant chemotherapy followed by radiation for subsequent progression, another underwent re-resection with mitral valve replacement, and another patient had salvage chemotherapy. For distant metastases, 1 patient underwent chemotherapy and radiation, and another had a small bowel metastasectomy at 2.7 years.

### Great Vessel Sarcoma

Eight patients with pulmonary artery sarcoma underwent 9 operations. Surgical techniques included pulmonary endarterectomy in 2 (22%), pulmonary artery resection in 5 (56%), pulmonary artery resection with pulmonary wedge resection in 1 (11%), pulmonary artery resection with pneumonectomy in 1 (11%), and re-resection in 1 (11%). Two patients (25%) were considered disease-free postoperatively. Adjuvant chemoradiotherapy was used following 5 (56%) resections, and adjuvant chemotherapy after 2 (22%). Fifteen recurrences were treated with pulmonary artery re-resection (n = 1), pulmonary wedge resection (n = 5), chemotherapy (n = 5), radiation therapy (n = 2), and distant metastasectomy (n = 2).

**Figure 4:**
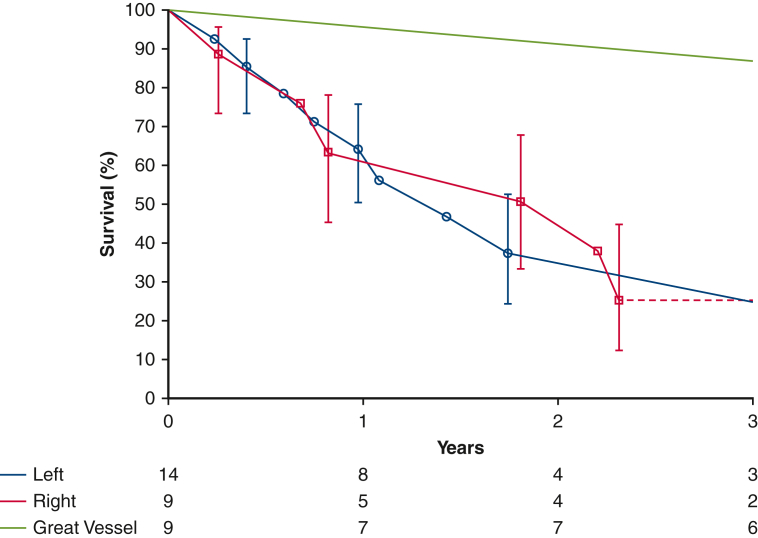
Survival estimated by Kaplan-Meier method in patients following surgical resection of cardiovascular sarcoma demonstrating left heart versus great vessel versus right heart location of the primary tumor. *Each symbol* represents a death, and *vertical bars* 68% confidence intervals equivalent to ±1 standard error. Number of patients remaining at risk is shown along the horizontal axis.

One patient was treated surgically for aortic sarcoma. The patient initially underwent surgery for an aortic arch thrombus that was found to be an intimal sarcoma, for which aortic arch resection with extra-anatomic ascending to descending aortic bypass was performed, as well as eighth rib metastasectomy. This patient was alive at 4.5 years with no evidence of disease progression. Great vessel involvement was associated with better survival than when the primary tumor occured in the heart (Figure 4).

## Discussion

### Principal Findings

Complete surgical resection of cardiovascular sarcomas remains the optimal treatment for these rare tumors because prior studies have shown that R0 resection improves median survival compared with incomplete resection.^5^, ^6^, ^7^, ^8^, ^9^, ^10^, ^11^ However, pursuit of complete resection must be balanced with preservation of residual cardiac function to prevent operative mortality. In our series, R0 resection was achieved in only a minority of patients (25%). However, there were no operative mortalities, and several patients underwent multimodality retreatment of recurrences and residual disease, which may prolong survival.^12^ The median survival of 3 years was similar to other published series, which range from 9 to 33 months.^3^, ^4^, ^5^, ^6^, ^7^, ^8^, ^9^, ^10^, ^11^, ^12^, ^13^, ^14^, ^15^, ^16^ Notably, even amongst the 8 patients who emerged disease-free from surgery, 4 (50%) developed recurrences during follow-up, illustrating the importance of rigorous follow-up and re-treatment of recurrences.

### Clinical Implications

The outcomes of this study highlight the persistently poor prognosis of patients with cardiac sarcoma, and the importance of advancing the treatment of this disease with aggressive innovation.

**Figure 5:**
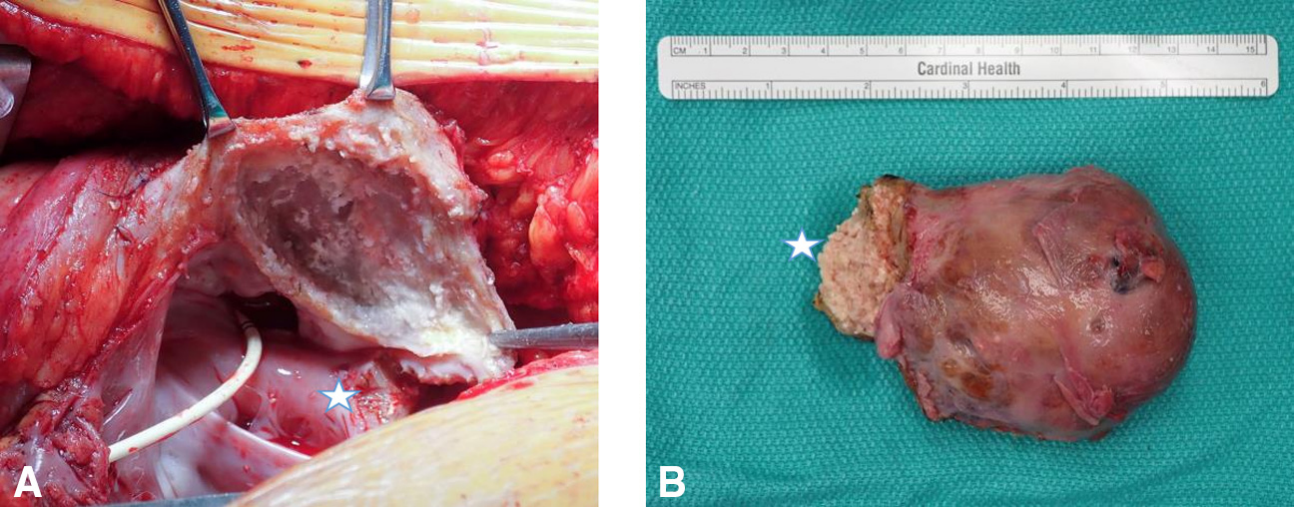
Intraoperative photographs after resection of a right atrial tumor demonstrating the tumor bed (A) and the resected specimen (B), which was causing flow obstruction through the right atrium but was fully resected with a gross margin off of its invasive stalk (*star*) following neoadjuvant chemoradiation. Final pathology showed no evidence of viable malignant cells.

Previous studies have investigated the role of neoadjuvant chemotherapy in increasing R0 resection; however, small sample sizes have precluded conclusive results.^11^, ^12^, ^13^, ^14^ In a recent case, 1 of our patients received neoadjuvant chemoradiation and underwent an R0 resection with no evidence of viable tumor in the specimen (Figure 5). Although long-term follow-up of this patient is pending, this promising finding suggests that in the subset of patients with bulky tumors without heart failure from tumor obstruction, neoadjuvant therapy may be an important adjunct to facilitate R0 resection. Use of neoadjuvant therapy requires a biopsy to establish the diagnosis. For many patients, a histologic diagnosis cannot be made percutaneously so surgery is necessary to confirm a diagnosis as well as relieving symptomatic and life-threatening obstruction. Last year we had a patient from out of state who was treated locally with neoadjuvant chemotherapy for sarcoma, but was found to have a melanoma after resection of the tumor. Some histologies have a poor response and are not likely to shrink appreciably. Systemic therapy may just delay surgery unnecessarily. Multidisciplinary collaboration between surgeon, oncologist, imaging cardiologist, and pathologist is critical to optimize patient selection for neoadjuvant therapy and guide multimodality treatment. We are currently likely to recommend adjuvant chemotherapy for most patients with cardiac tumors even with an R0 resection given the high likelihood for local recurrence.

With the current quality of imaging acquisition and processing, it is very unusual that we do not have a reasonable understanding about potential resectability (Video 1 demonstrates a case of a complex tumor resection). Excellent imaging allows for preoperative planning about what critical structures are at risk and the preparation of optimal exposure and potential reconstruction to handle those issues. In a recent case, we used computed tomography-based 3-dimensional (3D) printing to plan the complex resection of a sarcoma involving the fibrous skeleton of the heart (both right and left side tumor), with concomitant pulmonary embolectomy of tumor emboli. The 3D model helped us plan a novel surgical approach to the tumor using an incision through the aortic annulus at the right/noncoronary commissure, and to understand the precise location of pulmonary tumor emboli. For tumors that are difficult to expose or those with diffuse myocardial involvement, 3D printing may increase the likelihood of achieving an R0 resection by optimizing operative planning. The role for this technology should be investigated further. In an earlier era, this patient likely would have undergone biopsy and debulking, as was seen in 19% of the patients treated earlier in this series.

Endovascular therapies should also be considered as part of the arsenal for treating cardiovascular sarcomas. A recent patient with thoracoabdominal aortic sarcoma who was initially deemed unresectable developed a penetrating aortic ulcer following 5-Gy radiation therapy, subsequently underwent thoracic endovascular aortic repair followed by 50-Gy radiation, resulting in marked tumor regression and is still alive 9 years later.

### Limitations

This study is limited by the small number of patients due to the rarity of cardiac sarcoma even in a larger tertiary center. An exploration of the subject matter would be better if we included all those deemed inoperable, but we were unable to identify those patients and focused on the surgical experience. There was a high proportion of incomplete follow-up due to the large geographical referral base. Nonetheless, the heterogeneity in presentation and complexity of the disease allow even an observational study such as this to be instructive.

## Conclusions

Primary cardiovascular sarcoma is a rare, devastating disease, with a median survival of 3 years and frequent recurrences even in patients deemed free of disease after a complete R0 resection. In addition to striving for complete surgical resection, multidisciplinary teams must establish rigorous follow-up, with aggressive multimodality treatment of disease progression or recurrence to prolong survival. Further innovation, including multicenter collaborations to assess neoadjuvant therapy, improved imaging guided surgical planning (eg, 3D printing), and the use of other adjunctive treatments and technologies may improve outcomes.

### Conflict of Interest Statement

The authors reported no conflicts of interest.

The *Journal* policy requires editors and reviewers to disclose conflicts of interest and to decline handling or reviewing manuscripts for which they may have a conflict of interest. The editors and reviewers of this article have no conflicts of interest.

## Supplementary Data

Video 1 A case of recurrent left atrial myxoid liposarcoma resection and reconstruction. Video available at: https://www.jtcvs.org/article/S2666-2736(21)00271-0/fulltext.
