# Supplementary figures and images for: Outcomes after resection of primary cardiac sarcoma
Source: JTCVS Open. 2021 Sep 3;8:384–90. doi: 10.1016/j.xjon.2021.08.038 (PMC9390277; doi:10.1016/j.xjon.2021.08.038)

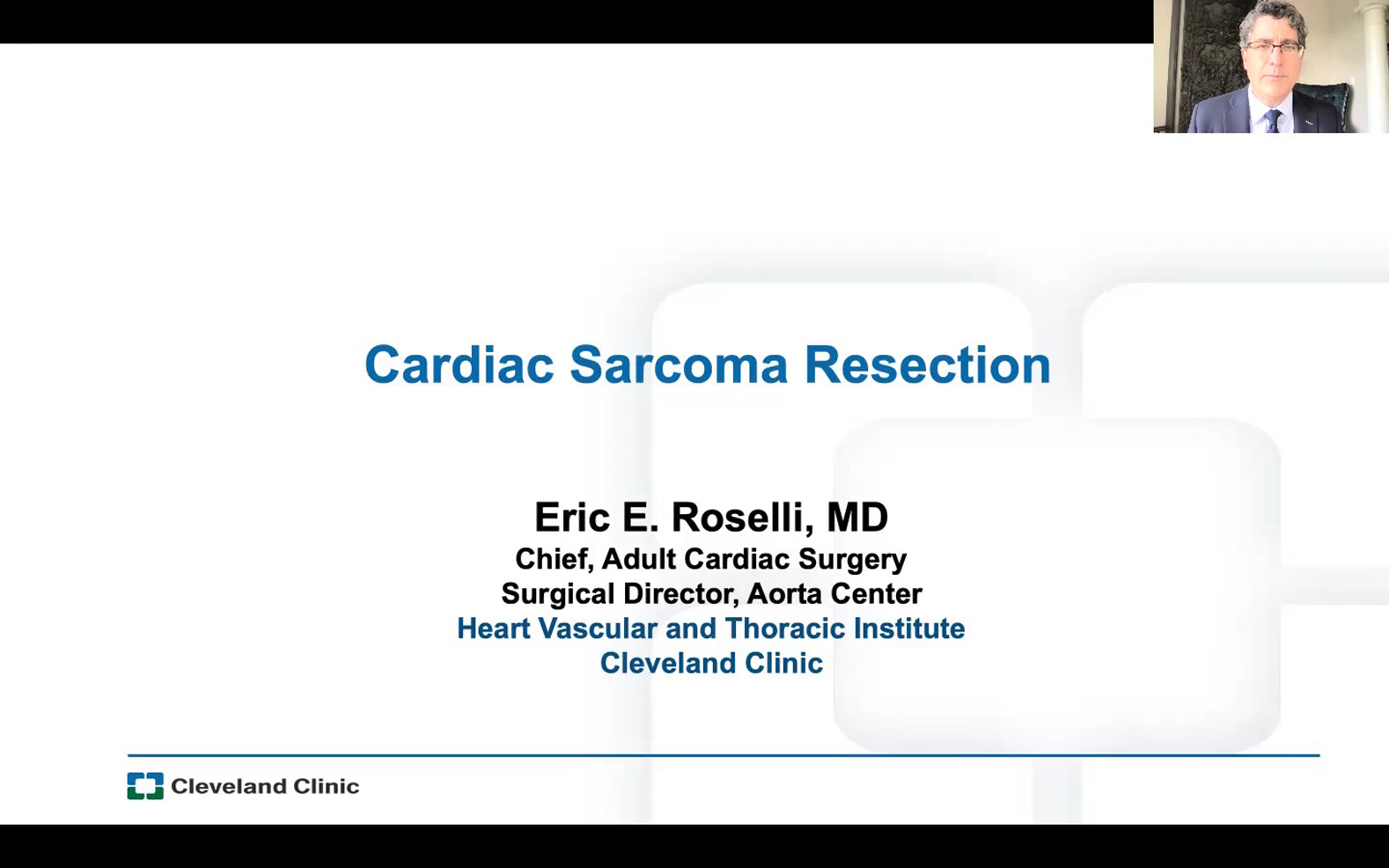

Supplement: Video 1 — A case of recurrent left atrial myxoid liposarcoma resection and reconstruction. Video available at: https://www.jtcvs.org/article/S2666-2736(21)00271-0/fulltext. [file fx2.jpg]
